# Strengthening Evidence Synthesis for Health Policymaking in Nepal: A New Fellowship Initiative

**DOI:** 10.3126/nje.v15i2.88516

**Published:** 2025-12-31

**Authors:** Padam Simkhada, Anju Vaidya, Pramod Regmi, Priya Paudyal, Edwin van Teijlingen, Meghnath Dhimal, Bikash Koirala, Akina Shrestha, Bibha Simkhada

**Affiliations:** 1 School of Human & Health Sciences, University of Huddersfield, Huddersfield, UK; 2 Faculty of Health & Medical Sciences, Bournemouth University, Bournemouth, UK; 3 Institute for Global Health & Wellbeing School of Medicine, Keele University, Keele, UK; 4 Nepal Health Research Council, Kathmandu, Nepal; 5 Green Tara Nepal, Kathmandu, Nepal; 6 Kathmandu University School of Medical Sciences, Nepal

## Introduction

The need for evidence-based policymaking has been widely recognised in the global health policy environment, including Nepal. However, in many countries, evidence-based policy and planning remain limited or even inadequate, largely due to a persistent disconnect between policymakers and researchers [1]. On several previous occasions, we have stressed the need to establish an Evidence Synthesis Centre in Nepal [2-3]. Such a Centre would require trained academics and researchers who are able to search, review, critically appraise and synthesise the most up-to-date literature [4]. The Centre is crucial for providing the best available evidence and informing decision-making for health and related policies and practice by (a) acting as a hub for synthesising research findings, (b) producing policy briefs tailored to the priority areas and decision-makers' needs, and (c) facilitating direct engagement between researchers and policymakers. Its role is especially important for addressing and mitigating emerging health challenges, as highlighted by our previous work during the recent COVID-19 pandemic [5].

This new Fellowship scheme is an international collaboration led by the University of Huddersfield in the UK, in close collaboration with Kathmandu University School of Medical Sciences (in Nepal), the Nepal Health Research Council, and several UK (United Kingdom) universities: Bournemouth University, the University of Sheffield, Canterbury Christ Church University, Keele University and the University of Chester. This unique Fellowship will start in the autumn of 2025 and is part of a larger project on strengthening institutional knowledge and capacity of federal, provincial and local governments for evidence-informed health policymaking in Nepal. This important initiative is funded by The British Academy and organisationally supported in the field by Green Tara Nepal.

This 12-month Fellowship programme will include several capacity strengthening training modules covering key aspects of e*vidence synthesis for policy, research and practice.* The overall aim is to establish a robust foundation of evidence-informed health policymaking in Nepal, thereby creating a sustainable mechanism for research into health policy and practice in the country. The programme aims to contribute to health policymaking in Nepal by strengthening the capacity of federal, provincial, and local governments to use research evidence in decision-making. We strongly believe that the Centre’s role will be crucial in generating high-quality evidence that directly informs policy and practice.

The applied programme aims to enhance the skills of 25 to 30 highly motivated academics, researchers and policy makers to conduct evidence synthesis, including systematic reviews and rapid evidence assessments, as well as develop knowledge and tools to translate evidence into clear policy briefs (see Box 1).

**Box 1: Main learning outcomes of the Fellowship programme**

By the end of this one-year programme, participants will:

- Understand the concept, barriers and challenges of evidence informed policy making.
- Understand the theory and application of different evidence synthesis methods.
- Design and register a systematic/scoping review protocol.
- Develop advanced skills in literature searching, screening, data extraction, and quality appraisal.
- Perform qualitative and quantitative synthesis.
- Use different tools for screening documents, appraising the quality of documents and managing the references.
- Prepare full draft of review manuscripts for publication.
- Be able to develop/prepare a policy-brief based on evidence

These Fellows will be mentored by national and international experts in evidence synthesis, social and political sciences, and getting evidence into policy. This Fellowship programme is good example of a South-North interdisciplinary collaboration benefitting from the experience and insights of individual staff and institution based in both high and low-income countries. The latter is important to ensure the learning is two ways, i.e. we learn from each other as well as ensuring that knowledge translation for evidence-informed decision-making is culturally appropriate, locally acceptable, and so on.

Therefore, this much-needed initiative brings together an interdisciplinary team [6] based at Nepal and UK-based institutions, aiming to enhance the use of high-quality research evidence to support effective health policy making in Nepal. We are optimistic that the programme will facilitate evidence-informed policymaking by strengthening the capacity of local researchers through well-supported training Fellowships, fostering a culture of evidence-based decision-making and evidence-informed policymaking to ensure long-term sustainability. It will enhance policymakers’ ability to access and utilise research evidence in their decision-making processes. Finally, we aim to provide an update of the Fellowship’s progress in a future editorial of the *Nepal Journal of Epidemiology*
